# Bis(4-amino­benzoato-κ*O*)triphenylanti­mony(V)

**DOI:** 10.1107/S1600536808032844

**Published:** 2008-10-18

**Authors:** Liyuan Wen, Handong Yin, Daqi Wang, Liansheng Cui, Minglei Yang

**Affiliations:** a College of Chemistry and Chemical Engineering, Liaocheng University, Shandong 252059, People’s Republic of China

## Abstract

The structure of the title compound, [Sb(C_6_H_5_)_3_(C_7_H_6_NO_2_)_2_], contains two independent mol­ecules of similar configuration. The Sb atoms exhibit a distorted trigonal–bipyramidal geometry with the O atoms of two carboxyl­ate groups in the axial positions and the C atoms of the phenyl groups in the equatorial positions. In the crystal structure, mol­ecules are connected by inter­molecular N—H⋯O and N—H⋯N hydrogen-bonding inter­actions forming a chain structure along [100].

## Related literature

For related structures, see: Wang *et al.* (2005 ▶).
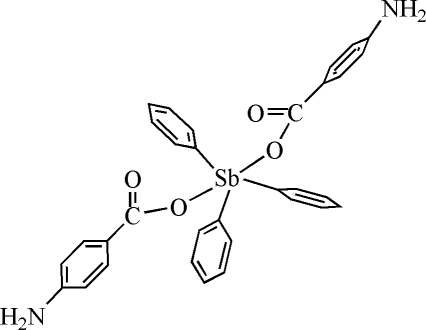


## Experimental

### 

#### Crystal data

[Sb(C_6_H_5_)_3_(C_7_H_6_NO_2_)_2_]
*M* *_r_* = 625.31
Monoclinic, 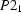

*a* = 9.2831 (11) Å
*b* = 18.971 (2) Å
*c* = 16.3868 (19) Å
β = 95.543 (2)°
*V* = 2872.3 (6) Å^3^
*Z* = 4
Mo *K*α radiation
μ = 1.00 mm^−1^
*T* = 298 (2) K
0.45 × 0.32 × 0.30 mm

#### Data collection

Siemens SMART CCD diffractometer
Absorption correction: multi-scan (*SADABS*; Sheldrick, 1996 ▶) *T*_min_ = 0.662, *T*_max_ = 0.754
14481 measured reflections
9132 independent reflections
7713 reflections with *I* > 2σ(*I*)
*R*_int_ = 0.024

#### Refinement

*R*[*F* ^2^ > 2σ(*F* ^2^)] = 0.040
*wR*(*F* ^2^) = 0.099
*S* = 1.00
9132 reflections
703 parameters
1 restraint
H-atom parameters constrained
Δρ_max_ = 0.62 e Å^−3^
Δρ_min_ = −0.63 e Å^−3^
Absolute structure: Flack (1983 ▶), 3905 Friedel pairs
Flack parameter: −0.01 (2)

### 

Data collection: *SMART* (Siemens, 1996 ▶); cell refinement: *SAINT* (Siemens, 1996 ▶); data reduction: *SAINT*; program(s) used to solve structure: *SHELXS97* (Sheldrick, 2008 ▶); program(s) used to refine structure: *SHELXL97* (Sheldrick, 2008 ▶); molecular graphics: *SHELXTL* (Sheldrick, 2008 ▶); software used to prepare material for publication: *SHELXTL*.

## Supplementary Material

Crystal structure: contains datablocks I, global. DOI: 10.1107/S1600536808032844/sg2267sup1.cif

Structure factors: contains datablocks I. DOI: 10.1107/S1600536808032844/sg2267Isup2.hkl

Additional supplementary materials:  crystallographic information; 3D view; checkCIF report

## Figures and Tables

**Table 1:** Hydrogen-bond geometry (Å, °)

*D*—H⋯*A*	*D*—H	H⋯*A*	*D*⋯*A*	*D*—H⋯*A*
N3—H3*B*⋯N2^i^	0.86	2.44	3.245 (11)	156
N3—H3*A*⋯O4^ii^	0.86	2.40	3.178 (8)	151
N2—H2*B*⋯O2^iii^	0.86	2.22	2.996 (8)	151
N1—H1*A*⋯O4^iv^	0.86	2.24	3.046 (8)	156

Symmetry codes: (i) 
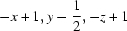
; (ii) 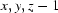; (iii) 
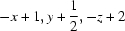
; (iv) 
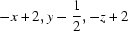
.

**Fig. 1.**
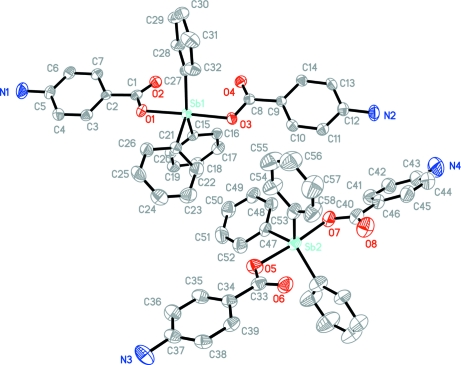
The molecular structure of the compound, showing 50% probability displacement ellipsoids. H atoms have been omitted for clarity.

**Fig. 2.**
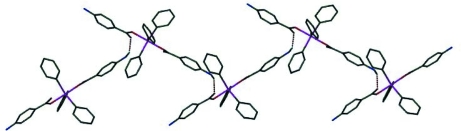
View of the one-dimensional extended chain structure in the title compound. Intermolecular hydrogen bonds are shown as dashed lines. H atoms are omitted.

## supplementary crystallographic information

### Comment

The organoantimony(V) derivatives have attracted considerable attention due to the significant antimicrobial properties as well as antitumor activities recently. As a part of our ongoing investigations in ths field we have synthesized the title compound and determined its crystal structure. The crystal structure of the title compound which contains two independent molecules is shown in Fig.1 The Sb atom in both molecules assumes a distorted trigonal bipyramidal coordination geometry, provided by two carboxylate groups at the axial positions and three phenyl groups at the equatorial positions. The Sb—O bond distances (Sb1—O1 = 2.091 (4) Å; Sb1—O3 = 2.114 (4) Å; Sb2—O5 = 2.098 (5) Å; Sb2—O7 = 2.114 (5) Å) are comparable to those found in organoantimony arylhydroxmates (Wang *et al.* 2005). The Sb—C bond distances (Sb1—C15 = 2.121 (6) Å; Sb1—C21 = 2.115 (5) Å; Sb1—C27 = 2.115 (6) Å; Sb2—C47 = 2.101 (7) Å; Sb2—C53 = 2.074 (8) Å; Sb2—C59 = 2.068 (8) Å) of the compound lie within the normal range for Sb—C (phenyl) bonds (2.10–2.13 Å). In the crystal packing, molecules are linked by intermolecular N—H···O hydrogen bonds (Fig.2, Table 1,)

### Experimental

The reaction was carried out under nitrogen atmosphere. 4- aminobenzoic acid (2 mmol) and sodium ethoxide (2.4 mmol) were added to a stirred solution of methanol (30 ml) in a Schlenk flask and stirred for 0.5 h. Triphenylantimony dichloride (1 mmol) was then added to the reactor and the reaction mixture was stirred for 12 h at room temperature. The resulting clear solution was evaporated under vacuum. The product was crystallized from dichloromethane/methanol (1:1) to yield colourless blocks (yield 86%. m.p.521 K). Anal. Calcd (%) for C_32_H_27_O_4_Sb_1_N_2_ (: C, 61.46; H, 4.35; O, 10.23; Sb, 19.47. Found (%): C, 61.40; H, 4.42; O, 10.28; Sb, 19.43

### Refinement

The C—H and N—H hydrogen atoms were positioned with idealized geometry and were refined isotropic with *U*_iso_(H) = 1.2 *U*_eq_(C, O) for all H atoms using a riding model with N—H = 0.86 Å and C—H = 0.93 Å.

### Crystal data

**Table:**

[Sb(C_6_H_5_)_3_(C_7_H_6_NO_2_)_2_]	*F*(000) = 1264
*M**_r_* = 625.31	*D*_x_ = 1.446 Mg m^−^^3^
Monoclinic, *P*2_1_	Mo *K*α radiation, λ = 0.71073 Å
*a* = 9.2831 (11) Å	Cell parameters from 6029 reflections
*b* = 18.971 (2) Å	θ = 2.4–25.0°
*c* = 16.3868 (19) Å	µ = 1.00 mm^−^^1^
β = 95.543 (2)°	*T* = 298 K
*V* = 2872.3 (6) Å^3^	Block, colourless
*Z* = 4	0.45 × 0.32 × 0.30 mm

### Data collection

**Table:**

Siemens SMART CCD diffractometer	9132 independent reflections
Radiation source: fine-focus sealed tube	7713 reflections with *I* > 2σ(*I*)
graphite	*R*_int_ = 0.024
φ and ω scans	θ_max_ = 25.0°, θ_min_ = 1.7°
Absorption correction: multi-scan (SADABS; Sheldrick, 1996)	*h* = −6→11
*T*_min_ = 0.662, *T*_max_ = 0.754	*k* = −22→21
14481 measured reflections	*l* = −18→19

### Refinement

**Table:**

Refinement on *F*^2^	Secondary atom site location: difference Fourier map
Least-squares matrix: full	Hydrogen site location: inferred from neighbouring sites
*R*[*F*^2^ > 2σ(*F*^2^)] = 0.040	H-atom parameters constrained
*wR*(*F*^2^) = 0.099	*w* = 1/[σ^2^(*F*_o_^2^) + (0.0504*P*)^2^ + 1.4161*P*] where *P* = (*F*_o_^2^ + 2*F*_c_^2^)/3
*S* = 1.00	(Δ/σ)_max_ = 0.020
9132 reflections	Δρ_max_ = 0.62 e Å^−^^3^
703 parameters	Δρ_min_ = −0.63 e Å^−^^3^
1 restraint	Absolute structure: Flack (1983), 3905 Friedel pairs
Primary atom site location: structure-invariant direct methods	Flack parameter: −0.01 (2)

### Fractional atomic coordinates and isotropic or equivalent isotropic displacement parameters (Å^2^)

**Table:**

	*x*	*y*	*z*	*U*_iso_*/*U*_eq_	
Sb1	0.73466 (4)	0.28424 (2)	0.82632 (2)	0.03634 (10)	
Sb2	0.18571 (5)	0.47931 (3)	0.51514 (3)	0.05668 (14)	
N1	1.1819 (8)	−0.0958 (4)	0.8817 (5)	0.087 (2)	
H1A	1.2195	−0.1147	0.9265	0.104*	
H1B	1.1936	−0.1151	0.8354	0.104*	
N2	0.2686 (7)	0.6590 (3)	0.8782 (4)	0.0706 (18)	
H2A	0.2283	0.6747	0.8324	0.085*	
H2B	0.2558	0.6804	0.9232	0.085*	
N3	0.5218 (8)	0.2962 (4)	0.1067 (4)	0.092 (2)	
H3A	0.5214	0.3139	0.0583	0.110*	
H3B	0.5542	0.2543	0.1163	0.110*	
N4	−0.2358 (11)	0.6832 (6)	0.8811 (6)	0.140 (4)	
H4A	−0.2671	0.6541	0.9156	0.168*	
H4B	−0.2497	0.7277	0.8863	0.168*	
O1	0.8572 (4)	0.1920 (2)	0.8234 (2)	0.0440 (9)	
O2	0.8288 (5)	0.1826 (2)	0.9549 (3)	0.0526 (11)	
O3	0.6149 (5)	0.3792 (2)	0.8218 (2)	0.0443 (9)	
O4	0.6343 (5)	0.3790 (2)	0.9567 (3)	0.0493 (10)	
O5	0.2755 (6)	0.4204 (3)	0.4243 (3)	0.0726 (13)	
O6	0.2597 (6)	0.5165 (4)	0.3472 (3)	0.0797 (16)	
O7	0.0979 (6)	0.5259 (3)	0.6166 (3)	0.0702 (13)	
O8	0.0856 (8)	0.6313 (4)	0.5604 (4)	0.100 (2)	
C1	0.8731 (6)	0.1581 (3)	0.8927 (4)	0.0435 (14)	
C2	0.9471 (6)	0.0893 (3)	0.8899 (3)	0.0389 (13)	
C3	0.9655 (7)	0.0577 (3)	0.8152 (4)	0.0508 (15)	
H3	0.9276	0.0791	0.7667	0.061*	
C4	1.0389 (7)	−0.0049 (4)	0.8120 (4)	0.0581 (17)	
H4	1.0457	−0.0272	0.7620	0.070*	
C5	1.1035 (7)	−0.0348 (4)	0.8843 (4)	0.0556 (16)	
C6	1.0839 (7)	−0.0032 (4)	0.9585 (4)	0.0564 (16)	
H6	1.1245	−0.0236	1.0070	0.068*	
C7	1.0065 (7)	0.0570 (3)	0.9614 (4)	0.0489 (14)	
H7	0.9929	0.0770	1.0119	0.059*	
C8	0.5971 (6)	0.4081 (3)	0.8911 (4)	0.0433 (13)	
C9	0.5226 (6)	0.4774 (3)	0.8866 (3)	0.0396 (12)	
C10	0.4583 (7)	0.5036 (3)	0.8128 (4)	0.0516 (15)	
H10	0.4709	0.4796	0.7645	0.062*	
C11	0.3775 (7)	0.5632 (4)	0.8093 (4)	0.0550 (16)	
H11	0.3378	0.5798	0.7586	0.066*	
C12	0.3531 (7)	0.5999 (3)	0.8797 (4)	0.0500 (15)	
C13	0.4238 (7)	0.5761 (3)	0.9538 (4)	0.0493 (15)	
H13	0.4159	0.6022	1.0012	0.059*	
C14	0.5039 (7)	0.5160 (3)	0.9582 (4)	0.0441 (14)	
H14	0.5464	0.5003	1.0087	0.053*	
C15	0.5510 (6)	0.2234 (3)	0.8487 (4)	0.0387 (13)	
C16	0.4431 (7)	0.2462 (4)	0.8938 (4)	0.0486 (15)	
H16	0.4476	0.2909	0.9172	0.058*	
C17	0.3272 (7)	0.2021 (4)	0.9040 (4)	0.0591 (17)	
H17	0.2544	0.2171	0.9351	0.071*	
C18	0.3189 (8)	0.1375 (4)	0.8694 (4)	0.0593 (17)	
H18	0.2408	0.1083	0.8771	0.071*	
C19	0.4247 (8)	0.1146 (4)	0.8232 (5)	0.0598 (17)	
H19	0.4176	0.0702	0.7992	0.072*	
C20	0.5409 (7)	0.1568 (4)	0.8123 (4)	0.0503 (16)	
H20	0.6127	0.1413	0.7807	0.060*	
C21	0.7352 (6)	0.2897 (4)	0.6975 (3)	0.0439 (12)	
C22	0.6235 (10)	0.3218 (5)	0.6514 (5)	0.074 (2)	
H22	0.5505	0.3441	0.6766	0.089*	
C23	0.6198 (11)	0.3206 (5)	0.5648 (5)	0.089 (2)	
H23	0.5430	0.3417	0.5332	0.106*	
C24	0.7258 (10)	0.2895 (6)	0.5277 (4)	0.079 (2)	
H24	0.7234	0.2896	0.4708	0.095*	
C25	0.8364 (10)	0.2580 (4)	0.5739 (5)	0.077 (2)	
H25	0.9106	0.2368	0.5485	0.092*	
C26	0.8399 (9)	0.2570 (4)	0.6583 (4)	0.0640 (19)	
H26	0.9148	0.2337	0.6890	0.077*	
C27	0.9160 (6)	0.3383 (3)	0.8840 (4)	0.0478 (14)	
C28	0.9866 (7)	0.3211 (4)	0.9585 (4)	0.0661 (18)	
H28	0.9509	0.2855	0.9899	0.079*	
C29	1.1109 (8)	0.3568 (4)	0.9870 (5)	0.076 (2)	
H29	1.1597	0.3437	1.0369	0.091*	
C30	1.1635 (8)	0.4102 (4)	0.9442 (5)	0.075 (2)	
H30	1.2470	0.4342	0.9642	0.090*	
C31	1.0905 (8)	0.4281 (5)	0.8708 (5)	0.083 (2)	
H31	1.1257	0.4648	0.8407	0.100*	
C32	0.9654 (8)	0.3932 (4)	0.8396 (4)	0.0664 (18)	
H32	0.9164	0.4068	0.7899	0.080*	
C33	0.2923 (8)	0.4546 (5)	0.3556 (4)	0.0627 (17)	
C34	0.3543 (8)	0.4121 (4)	0.2917 (4)	0.0572 (16)	
C35	0.4020 (9)	0.3444 (5)	0.3069 (4)	0.0703 (19)	
H35	0.3943	0.3240	0.3578	0.084*	
C36	0.4620 (9)	0.3065 (5)	0.2458 (5)	0.077 (2)	
H36	0.4967	0.2612	0.2571	0.092*	
C37	0.4709 (9)	0.3350 (5)	0.1685 (4)	0.0633 (19)	
C38	0.4184 (8)	0.4026 (5)	0.1544 (4)	0.0637 (18)	
H38	0.4218	0.4225	0.1027	0.076*	
C39	0.3612 (8)	0.4412 (4)	0.2145 (4)	0.0650 (18)	
H39	0.3274	0.4867	0.2035	0.078*	
C40	0.0651 (9)	0.5920 (5)	0.6165 (5)	0.0720 (18)	
C41	−0.0088 (9)	0.6170 (4)	0.6880 (5)	0.0703 (19)	
C42	−0.0629 (9)	0.5693 (5)	0.7415 (5)	0.080 (2)	
H42	−0.0477	0.5214	0.7335	0.097*	
C43	−0.1372 (10)	0.5896 (5)	0.8051 (5)	0.086 (2)	
H43	−0.1696	0.5555	0.8398	0.104*	
C44	−0.1652 (12)	0.6590 (6)	0.8191 (6)	0.095 (3)	
C45	−0.1070 (12)	0.7097 (6)	0.7685 (7)	0.106 (3)	
H45	−0.1176	0.7575	0.7790	0.127*	
C46	−0.0299 (11)	0.6869 (5)	0.6996 (6)	0.093 (2)	
H46	0.0042	0.7198	0.6641	0.112*	
C47	0.2041 (8)	0.3860 (4)	0.5844 (4)	0.0598 (16)	
C48	0.2146 (8)	0.3879 (4)	0.6682 (4)	0.0654 (18)	
H48	0.2099	0.4312	0.6945	0.079*	
C49	0.2320 (10)	0.3280 (5)	0.7144 (5)	0.079 (2)	
H49	0.2359	0.3310	0.7712	0.095*	
C50	0.2434 (10)	0.2643 (5)	0.6785 (6)	0.087 (2)	
H50	0.2643	0.2244	0.7106	0.104*	
C51	0.2243 (12)	0.2586 (5)	0.5953 (6)	0.096 (3)	
H51	0.2218	0.2145	0.5705	0.115*	
C52	0.2082 (10)	0.3211 (5)	0.5465 (5)	0.079 (2)	
H52	0.2005	0.3180	0.4896	0.095*	
C53	0.3729 (8)	0.5391 (4)	0.5342 (5)	0.0674 (18)	
C54	0.4865 (9)	0.5096 (5)	0.5823 (6)	0.102 (3)	
H54	0.4787	0.4639	0.6019	0.122*	
C55	0.6130 (12)	0.5475 (6)	0.6017 (8)	0.126 (3)	
H55	0.6863	0.5279	0.6373	0.151*	
C56	0.6326 (12)	0.6126 (6)	0.5702 (7)	0.122 (3)	
H56	0.7192	0.6369	0.5820	0.147*	
C57	0.5214 (11)	0.6414 (6)	0.5206 (8)	0.131 (3)	
H57	0.5307	0.6864	0.4992	0.158*	
C58	0.3939 (10)	0.6036 (5)	0.5018 (7)	0.104 (3)	
H58	0.3210	0.6231	0.4659	0.125*	
C59	−0.0113 (9)	0.4935 (5)	0.4468 (5)	0.083 (2)	
C60	−0.1083 (11)	0.4402 (6)	0.4473 (7)	0.128 (3)	
H60	−0.0815	0.3977	0.4727	0.154*	
C61	−0.2491 (12)	0.4491 (7)	0.4096 (9)	0.151 (4)	
H61	−0.3161	0.4129	0.4115	0.181*	
C62	−0.2886 (12)	0.5100 (6)	0.3702 (7)	0.121 (3)	
H62	−0.3837	0.5164	0.3479	0.145*	
C63	−0.1893 (10)	0.5617 (6)	0.3636 (7)	0.117 (3)	
H63	−0.2121	0.6018	0.3324	0.140*	
C64	−0.0543 (10)	0.5531 (5)	0.4043 (6)	0.095 (3)	
H64	0.0118	0.5898	0.4028	0.113*	

### Atomic displacement parameters (Å^2^)

**Table:**

	*U*^11^	*U*^22^	*U*^33^	*U*^12^	*U*^13^	*U*^23^
Sb1	0.03271 (18)	0.03578 (18)	0.04090 (19)	−0.0036 (2)	0.00541 (14)	−0.00028 (19)
Sb2	0.0501 (3)	0.0742 (3)	0.0460 (2)	0.0103 (2)	0.0064 (2)	0.0135 (2)
N1	0.092 (5)	0.080 (5)	0.087 (5)	0.040 (4)	0.004 (4)	0.007 (4)
N2	0.080 (4)	0.060 (4)	0.073 (4)	0.027 (4)	0.013 (3)	−0.006 (3)
N3	0.128 (6)	0.080 (5)	0.072 (4)	−0.019 (5)	0.040 (4)	−0.015 (4)
N4	0.154 (8)	0.151 (8)	0.118 (7)	0.053 (7)	0.030 (6)	−0.033 (6)
O1	0.035 (2)	0.041 (2)	0.056 (2)	0.0056 (19)	0.0067 (18)	0.0095 (19)
O2	0.050 (3)	0.053 (3)	0.056 (3)	−0.004 (2)	0.008 (2)	−0.002 (2)
O3	0.045 (2)	0.038 (2)	0.051 (2)	0.0028 (19)	0.0074 (19)	−0.0051 (18)
O4	0.047 (2)	0.043 (2)	0.057 (3)	−0.002 (2)	0.000 (2)	0.005 (2)
O5	0.078 (3)	0.095 (3)	0.048 (2)	−0.001 (3)	0.018 (2)	0.005 (2)
O6	0.082 (4)	0.097 (4)	0.060 (3)	0.027 (3)	0.010 (3)	0.010 (3)
O7	0.071 (3)	0.073 (3)	0.068 (3)	0.020 (3)	0.014 (2)	0.013 (2)
O8	0.109 (5)	0.103 (5)	0.089 (4)	0.017 (4)	0.028 (4)	0.035 (4)
C1	0.027 (3)	0.049 (3)	0.053 (3)	−0.011 (3)	0.001 (3)	0.001 (3)
C2	0.031 (3)	0.040 (3)	0.044 (3)	−0.004 (3)	−0.003 (2)	0.002 (3)
C3	0.050 (3)	0.047 (3)	0.054 (3)	−0.001 (3)	−0.003 (3)	0.006 (3)
C4	0.062 (4)	0.053 (4)	0.058 (3)	0.008 (3)	0.001 (3)	−0.004 (3)
C5	0.051 (3)	0.053 (4)	0.064 (4)	0.010 (3)	0.008 (3)	0.011 (3)
C6	0.056 (4)	0.059 (4)	0.054 (3)	0.010 (3)	0.001 (3)	0.019 (3)
C7	0.045 (3)	0.053 (3)	0.049 (3)	0.000 (3)	0.002 (3)	0.008 (3)
C8	0.034 (3)	0.039 (3)	0.057 (3)	−0.009 (3)	0.004 (3)	−0.005 (3)
C9	0.031 (3)	0.039 (3)	0.049 (3)	−0.004 (3)	0.002 (2)	−0.006 (3)
C10	0.058 (4)	0.052 (4)	0.045 (3)	0.006 (3)	0.005 (3)	−0.009 (3)
C11	0.056 (4)	0.058 (4)	0.049 (3)	0.009 (3)	−0.002 (3)	0.000 (3)
C12	0.050 (3)	0.046 (4)	0.055 (4)	0.002 (3)	0.013 (3)	0.000 (3)
C13	0.053 (3)	0.044 (3)	0.052 (3)	−0.002 (3)	0.011 (3)	−0.013 (3)
C14	0.044 (3)	0.040 (3)	0.048 (3)	−0.004 (3)	0.005 (3)	−0.003 (3)
C15	0.035 (3)	0.036 (3)	0.045 (3)	−0.006 (3)	0.000 (2)	0.007 (2)
C16	0.044 (3)	0.050 (3)	0.053 (3)	0.000 (3)	0.006 (3)	0.000 (3)
C17	0.043 (3)	0.071 (4)	0.066 (4)	−0.008 (3)	0.017 (3)	0.011 (3)
C18	0.050 (4)	0.052 (4)	0.076 (4)	−0.019 (3)	0.003 (3)	0.009 (3)
C19	0.050 (4)	0.044 (4)	0.082 (4)	−0.018 (3)	−0.006 (3)	−0.003 (3)
C20	0.043 (3)	0.049 (4)	0.058 (3)	−0.004 (3)	0.000 (3)	−0.004 (3)
C21	0.046 (3)	0.049 (3)	0.039 (3)	−0.001 (3)	0.011 (2)	0.003 (3)
C22	0.080 (4)	0.086 (5)	0.059 (4)	0.020 (4)	0.015 (4)	0.017 (4)
C23	0.099 (5)	0.106 (6)	0.060 (4)	0.016 (5)	−0.001 (4)	0.017 (4)
C24	0.098 (5)	0.092 (5)	0.049 (4)	−0.005 (5)	0.022 (4)	0.008 (4)
C25	0.084 (5)	0.089 (5)	0.061 (4)	−0.001 (4)	0.029 (4)	−0.017 (4)
C26	0.068 (4)	0.070 (4)	0.054 (4)	0.010 (4)	0.009 (3)	0.000 (3)
C27	0.031 (3)	0.046 (3)	0.066 (4)	−0.007 (3)	0.002 (3)	−0.014 (3)
C28	0.059 (4)	0.054 (4)	0.083 (4)	−0.013 (3)	−0.009 (4)	−0.009 (3)
C29	0.060 (4)	0.065 (5)	0.097 (5)	−0.009 (4)	−0.022 (4)	−0.005 (4)
C30	0.039 (4)	0.078 (5)	0.107 (5)	−0.012 (4)	0.001 (4)	−0.026 (4)
C31	0.079 (5)	0.082 (5)	0.093 (5)	−0.048 (4)	0.034 (4)	−0.011 (4)
C32	0.061 (4)	0.069 (4)	0.069 (4)	−0.028 (4)	0.010 (3)	−0.002 (3)
C33	0.052 (4)	0.083 (4)	0.053 (4)	0.000 (4)	0.005 (3)	0.010 (3)
C34	0.055 (4)	0.075 (4)	0.043 (3)	−0.006 (3)	0.009 (3)	0.001 (3)
C35	0.085 (4)	0.084 (5)	0.043 (3)	−0.008 (4)	0.013 (3)	0.004 (3)
C36	0.082 (5)	0.082 (5)	0.069 (4)	−0.005 (4)	0.017 (4)	0.003 (4)
C37	0.066 (4)	0.078 (5)	0.048 (4)	−0.016 (4)	0.013 (3)	−0.005 (3)
C38	0.059 (4)	0.092 (5)	0.042 (3)	−0.013 (4)	0.013 (3)	0.005 (3)
C39	0.049 (4)	0.087 (5)	0.059 (4)	0.002 (3)	0.005 (3)	0.016 (3)
C40	0.063 (4)	0.080 (4)	0.074 (4)	0.010 (4)	0.009 (3)	0.013 (4)
C41	0.068 (4)	0.069 (4)	0.073 (4)	0.019 (4)	0.002 (4)	−0.004 (4)
C42	0.076 (4)	0.091 (5)	0.076 (4)	0.013 (4)	0.013 (4)	0.001 (4)
C43	0.095 (5)	0.097 (5)	0.069 (4)	0.016 (5)	0.017 (4)	−0.005 (4)
C44	0.098 (6)	0.106 (6)	0.081 (5)	0.021 (5)	0.008 (5)	−0.016 (5)
C45	0.111 (6)	0.096 (6)	0.107 (6)	0.027 (5)	0.000 (5)	−0.015 (5)
C46	0.098 (5)	0.087 (5)	0.093 (5)	0.024 (5)	0.002 (4)	0.009 (4)
C47	0.059 (3)	0.063 (4)	0.059 (3)	0.015 (3)	0.012 (3)	0.012 (3)
C48	0.076 (4)	0.070 (4)	0.051 (4)	0.014 (4)	0.007 (3)	0.010 (3)
C49	0.082 (5)	0.082 (5)	0.074 (5)	0.019 (4)	0.010 (4)	0.025 (4)
C50	0.097 (5)	0.080 (6)	0.085 (5)	0.016 (4)	0.016 (4)	0.023 (4)
C51	0.111 (6)	0.076 (5)	0.102 (6)	0.021 (5)	0.013 (5)	−0.007 (4)
C52	0.092 (5)	0.080 (5)	0.068 (4)	0.006 (4)	0.019 (4)	0.002 (4)
C53	0.058 (4)	0.075 (4)	0.071 (4)	0.011 (3)	0.015 (3)	0.014 (3)
C54	0.079 (5)	0.109 (5)	0.113 (5)	−0.009 (5)	−0.016 (5)	0.023 (5)
C55	0.095 (6)	0.136 (7)	0.142 (7)	−0.018 (6)	−0.021 (6)	0.026 (6)
C56	0.093 (6)	0.125 (7)	0.144 (7)	−0.027 (6)	−0.008 (6)	0.007 (6)
C57	0.094 (6)	0.114 (7)	0.186 (8)	−0.017 (6)	0.011 (6)	0.018 (7)
C58	0.072 (5)	0.097 (5)	0.140 (6)	0.001 (5)	−0.004 (5)	0.020 (5)
C59	0.067 (4)	0.110 (5)	0.073 (4)	−0.005 (4)	0.009 (3)	0.028 (4)
C60	0.108 (6)	0.130 (6)	0.138 (6)	−0.024 (5)	−0.031 (5)	0.053 (5)
C61	0.101 (7)	0.167 (8)	0.178 (8)	−0.022 (7)	−0.016 (6)	0.062 (7)
C62	0.084 (6)	0.152 (8)	0.122 (6)	0.004 (6)	−0.013 (5)	0.042 (6)
C63	0.109 (6)	0.126 (7)	0.108 (6)	0.026 (6)	−0.021 (6)	0.006 (6)
C64	0.088 (5)	0.098 (5)	0.090 (5)	0.018 (5)	−0.029 (4)	0.004 (4)

### Geometric parameters (Å, °)

**Table:**

Sb1—O1	2.091 (4)	C24—H24	0.9300
Sb1—C21	2.115 (5)	C25—C26	1.381 (10)
Sb1—O3	2.114 (4)	C25—H25	0.9300
Sb1—C27	2.115 (6)	C26—H26	0.9300
Sb1—C15	2.121 (6)	C27—C32	1.375 (7)
Sb2—C59	2.068 (8)	C27—C28	1.368 (7)
Sb2—O5	2.098 (5)	C28—C29	1.379 (7)
Sb2—C47	2.101 (7)	C28—H28	0.9300
Sb2—C53	2.074 (8)	C29—C30	1.351 (8)
Sb2—O7	2.114 (5)	C29—H29	0.9300
N1—C5	1.369 (9)	C30—C31	1.364 (8)
N1—H1A	0.8600	C30—H30	0.9300
N1—H1B	0.8600	C31—C32	1.390 (7)
N2—C12	1.367 (8)	C31—H31	0.9300
N2—H2A	0.8600	C32—H32	0.9300
N2—H2B	0.8600	C33—C34	1.481 (11)
N3—C37	1.373 (10)	C34—C35	1.372 (11)
N3—H3A	0.8600	C34—C39	1.388 (9)
N3—H3B	0.8600	C35—C36	1.391 (11)
N4—C44	1.341 (12)	C35—H35	0.9300
N4—H4A	0.8600	C36—C37	1.388 (10)
N4—H4B	0.8600	C36—H36	0.9300
O1—C1	1.300 (7)	C37—C38	1.382 (11)
O2—C1	1.227 (7)	C38—C39	1.375 (11)
O3—C8	1.287 (7)	C38—H38	0.9300
O4—C8	1.228 (7)	C39—H39	0.9300
O5—C33	1.320 (9)	C40—C41	1.491 (11)
O6—C33	1.219 (9)	C41—C46	1.357 (12)
O7—C40	1.290 (10)	C41—C42	1.386 (12)
O8—C40	1.212 (9)	C42—C43	1.360 (11)
C1—C2	1.478 (9)	C42—H42	0.9300
C2—C3	1.389 (9)	C43—C44	1.366 (14)
C2—C7	1.388 (8)	C43—H43	0.9300
C3—C4	1.372 (9)	C44—C45	1.411 (15)
C3—H3	0.9300	C45—C46	1.459 (13)
C4—C5	1.395 (9)	C45—H45	0.9300
C4—H4	0.9300	C46—H46	0.9300
C5—C6	1.384 (9)	C47—C48	1.367 (9)
C6—C7	1.352 (9)	C47—C52	1.381 (11)
C6—H6	0.9300	C48—C49	1.367 (11)
C7—H7	0.9300	C48—H48	0.9300
C8—C9	1.483 (9)	C49—C50	1.353 (13)
C9—C10	1.388 (8)	C49—H49	0.9300
C9—C14	1.409 (8)	C50—C51	1.362 (13)
C10—C11	1.356 (9)	C50—H50	0.9300
C10—H10	0.9300	C51—C52	1.430 (12)
C11—C12	1.385 (9)	C51—H51	0.9300
C11—H11	0.9300	C52—H52	0.9300
C12—C13	1.397 (9)	C53—C58	1.354 (8)
C13—C14	1.360 (8)	C53—C54	1.374 (8)
C13—H13	0.9300	C54—C55	1.388 (8)
C14—H14	0.9300	C54—H54	0.9300
C15—C16	1.370 (9)	C55—C56	1.357 (9)
C15—C20	1.396 (9)	C55—H55	0.9300
C16—C17	1.386 (9)	C56—C57	1.365 (9)
C16—H16	0.9300	C56—H56	0.9300
C17—C18	1.349 (10)	C57—C58	1.393 (8)
C17—H17	0.9300	C57—H57	0.9300
C18—C19	1.367 (10)	C58—H58	0.9300
C18—H18	0.9300	C59—C60	1.355 (8)
C19—C20	1.370 (9)	C59—C64	1.367 (8)
C19—H19	0.9300	C60—C61	1.401 (8)
C20—H20	0.9300	C60—H60	0.9300
C21—C22	1.365 (10)	C61—C62	1.357 (8)
C21—C26	1.364 (9)	C61—H61	0.9300
C22—C23	1.416 (11)	C62—C63	1.357 (9)
C22—H22	0.9300	C62—H62	0.9300
C23—C24	1.342 (12)	C63—C64	1.371 (8)
C23—H23	0.9300	C63—H63	0.9300
C24—C25	1.354 (12)	C64—H64	0.9300
			
O1—Sb1—C21	88.0 (2)	C32—C27—C28	119.8 (6)
O1—Sb1—O3	176.32 (17)	C32—C27—Sb1	115.0 (5)
C21—Sb1—O3	88.6 (2)	C28—C27—Sb1	125.2 (5)
O1—Sb1—C27	90.3 (2)	C27—C28—C29	119.9 (7)
C21—Sb1—C27	110.1 (2)	C27—C28—H28	120.1
O3—Sb1—C27	89.8 (2)	C29—C28—H28	120.1
O1—Sb1—C15	89.8 (2)	C30—C29—C28	121.7 (7)
C21—Sb1—C15	106.2 (2)	C30—C29—H29	119.2
O3—Sb1—C15	92.4 (2)	C28—C29—H29	119.2
C27—Sb1—C15	143.6 (2)	C29—C30—C31	118.0 (7)
C59—Sb2—O5	94.4 (3)	C29—C30—H30	121.0
C59—Sb2—C47	115.1 (3)	C31—C30—H30	121.0
O5—Sb2—C47	85.3 (2)	C30—C31—C32	122.2 (7)
C59—Sb2—C53	134.0 (3)	C30—C31—H31	118.9
O5—Sb2—C53	90.8 (3)	C32—C31—H31	118.9
C47—Sb2—C53	110.9 (3)	C27—C32—C31	118.4 (7)
C59—Sb2—O7	89.0 (3)	C27—C32—H32	120.8
O5—Sb2—O7	172.1 (2)	C31—C32—H32	120.8
C47—Sb2—O7	86.9 (2)	O6—C33—O5	121.5 (7)
C53—Sb2—O7	91.9 (3)	O6—C33—C34	123.6 (7)
C5—N1—H1A	120.0	O5—C33—C34	114.9 (8)
C5—N1—H1B	120.0	C35—C34—C39	119.6 (7)
H1A—N1—H1B	120.0	C35—C34—C33	121.5 (6)
C12—N2—H2A	120.0	C39—C34—C33	118.9 (7)
C12—N2—H2B	120.0	C34—C35—C36	119.9 (7)
H2A—N2—H2B	120.0	C34—C35—H35	120.1
C37—N3—H3A	120.0	C36—C35—H35	120.1
C37—N3—H3B	120.0	C37—C36—C35	121.2 (8)
H3A—N3—H3B	120.0	C37—C36—H36	119.4
C44—N4—H4A	120.0	C35—C36—H36	119.4
C44—N4—H4B	120.0	N3—C37—C38	121.0 (7)
H4A—N4—H4B	120.0	N3—C37—C36	121.3 (8)
C1—O1—Sb1	114.2 (4)	C38—C37—C36	117.6 (7)
C8—O3—Sb1	116.4 (4)	C37—C38—C39	121.9 (7)
C33—O5—Sb2	115.5 (5)	C37—C38—H38	119.1
C40—O7—Sb2	121.0 (5)	C39—C38—H38	119.1
O2—C1—O1	121.2 (6)	C34—C39—C38	119.7 (8)
O2—C1—C2	123.7 (6)	C34—C39—H39	120.1
O1—C1—C2	115.1 (5)	C38—C39—H39	120.1
C3—C2—C7	118.8 (6)	O8—C40—O7	122.9 (8)
C3—C2—C1	120.3 (5)	O8—C40—C41	121.2 (8)
C7—C2—C1	120.8 (6)	O7—C40—C41	115.7 (7)
C4—C3—C2	120.6 (6)	C46—C41—C42	118.9 (8)
C4—C3—H3	119.7	C46—C41—C40	120.2 (8)
C2—C3—H3	119.7	C42—C41—C40	120.8 (8)
C3—C4—C5	119.7 (6)	C43—C42—C41	122.7 (9)
C3—C4—H4	120.1	C43—C42—H42	118.6
C5—C4—H4	120.1	C41—C42—H42	118.6
N1—C5—C6	120.5 (6)	C44—C43—C42	121.4 (10)
N1—C5—C4	120.3 (7)	C44—C43—H43	119.3
C6—C5—C4	119.1 (6)	C42—C43—H43	119.3
C7—C6—C5	120.8 (6)	N4—C44—C43	124.9 (11)
C7—C6—H6	119.6	N4—C44—C45	117.1 (11)
C5—C6—H6	119.6	C43—C44—C45	117.9 (10)
C6—C7—C2	120.8 (6)	C44—C45—C46	119.8 (10)
C6—C7—H7	119.6	C44—C45—H45	120.1
C2—C7—H7	119.6	C46—C45—H45	120.1
O4—C8—O3	122.1 (6)	C41—C46—C45	119.1 (10)
O4—C8—C9	122.2 (6)	C41—C46—H46	120.4
O3—C8—C9	115.7 (6)	C45—C46—H46	120.4
C10—C9—C14	117.5 (6)	C48—C47—C52	118.3 (7)
C10—C9—C8	121.3 (5)	C48—C47—Sb2	120.9 (6)
C14—C9—C8	120.9 (5)	C52—C47—Sb2	120.8 (6)
C11—C10—C9	121.7 (6)	C47—C48—C49	121.8 (8)
C11—C10—H10	119.1	C47—C48—H48	119.1
C9—C10—H10	119.1	C49—C48—H48	119.1
C10—C11—C12	121.2 (6)	C50—C49—C48	120.8 (8)
C10—C11—H11	119.4	C50—C49—H49	119.6
C12—C11—H11	119.4	C48—C49—H49	119.6
N2—C12—C11	122.4 (6)	C49—C50—C51	119.8 (8)
N2—C12—C13	120.0 (6)	C49—C50—H50	120.1
C11—C12—C13	117.5 (6)	C51—C50—H50	120.1
C14—C13—C12	121.7 (6)	C50—C51—C52	119.4 (8)
C14—C13—H13	119.2	C50—C51—H51	120.3
C12—C13—H13	119.2	C52—C51—H51	120.3
C13—C14—C9	120.2 (6)	C47—C52—C51	119.5 (8)
C13—C14—H14	119.9	C47—C52—H52	120.2
C9—C14—H14	119.9	C51—C52—H52	120.2
C16—C15—C20	119.6 (6)	C58—C53—C54	117.5 (8)
C16—C15—Sb1	124.3 (5)	C58—C53—Sb2	125.8 (6)
C20—C15—Sb1	116.1 (5)	C54—C53—Sb2	116.7 (6)
C17—C16—C15	119.4 (6)	C53—C54—C55	120.4 (9)
C17—C16—H16	120.3	C53—C54—H54	119.8
C15—C16—H16	120.3	C55—C54—H54	119.8
C18—C17—C16	120.7 (7)	C56—C55—C54	121.7 (11)
C18—C17—H17	119.6	C56—C55—H55	119.1
C16—C17—H17	119.6	C54—C55—H55	119.1
C17—C18—C19	120.4 (6)	C55—C56—C57	118.0 (11)
C17—C18—H18	119.8	C55—C56—H56	121.0
C19—C18—H18	119.8	C57—C56—H56	121.0
C20—C19—C18	120.3 (7)	C56—C57—C58	120.2 (11)
C20—C19—H19	119.9	C56—C57—H57	119.9
C18—C19—H19	119.9	C58—C57—H57	119.9
C19—C20—C15	119.6 (7)	C53—C58—C57	122.0 (10)
C19—C20—H20	120.2	C53—C58—H58	119.0
C15—C20—H20	120.2	C57—C58—H58	119.0
C22—C21—C26	118.7 (6)	C60—C59—C64	117.3 (9)
C22—C21—Sb1	119.9 (5)	C60—C59—Sb2	117.0 (6)
C26—C21—Sb1	121.2 (5)	C64—C59—Sb2	125.7 (7)
C21—C22—C23	119.4 (8)	C59—C60—C61	120.0 (10)
C21—C22—H22	120.3	C59—C60—H60	120.0
C23—C22—H22	120.3	C61—C60—H60	120.0
C24—C23—C22	120.8 (8)	C62—C61—C60	120.7 (11)
C24—C23—H23	119.6	C62—C61—H61	119.6
C22—C23—H23	119.6	C60—C61—H61	119.6
C23—C24—C25	119.3 (7)	C61—C62—C63	119.9 (11)
C23—C24—H24	120.3	C61—C62—H62	120.1
C25—C24—H24	120.3	C63—C62—H62	120.1
C24—C25—C26	120.6 (8)	C62—C63—C64	118.1 (11)
C24—C25—H25	119.7	C62—C63—H63	120.9
C26—C25—H25	119.7	C64—C63—H63	120.9
C25—C26—C21	121.1 (7)	C59—C64—C63	123.7 (10)
C25—C26—H26	119.5	C59—C64—H64	118.2
C21—C26—H26	119.5	C63—C64—H64	118.2
			
C21—Sb1—O1—C1	−170.7 (4)	C32—C27—C28—C29	−3.1 (11)
O3—Sb1—O1—C1	170 (2)	Sb1—C27—C28—C29	174.8 (6)
C27—Sb1—O1—C1	79.2 (4)	C27—C28—C29—C30	2.1 (13)
C15—Sb1—O1—C1	−64.4 (4)	C28—C29—C30—C31	−0.5 (13)
O1—Sb1—O3—C8	−152 (3)	C29—C30—C31—C32	0.1 (13)
C21—Sb1—O3—C8	−171.1 (4)	C28—C27—C32—C31	2.6 (11)
C27—Sb1—O3—C8	−60.9 (4)	Sb1—C27—C32—C31	−175.4 (6)
C15—Sb1—O3—C8	82.7 (4)	C30—C31—C32—C27	−1.1 (13)
C59—Sb2—O5—C33	64.5 (5)	Sb2—O5—C33—O6	1.3 (10)
C47—Sb2—O5—C33	179.3 (5)	Sb2—O5—C33—C34	−179.4 (5)
C53—Sb2—O5—C33	−69.8 (5)	O6—C33—C34—C35	173.7 (8)
O7—Sb2—O5—C33	−179.9 (15)	O5—C33—C34—C35	−5.6 (11)
C59—Sb2—O7—C40	−68.5 (6)	O6—C33—C34—C39	−7.6 (11)
O5—Sb2—O7—C40	175.6 (14)	O5—C33—C34—C39	173.1 (6)
C47—Sb2—O7—C40	176.4 (6)	C39—C34—C35—C36	2.4 (12)
C53—Sb2—O7—C40	65.5 (6)	C33—C34—C35—C36	−178.9 (7)
Sb1—O1—C1—O2	−5.7 (7)	C34—C35—C36—C37	−1.9 (13)
Sb1—O1—C1—C2	174.4 (4)	C35—C36—C37—N3	−176.0 (8)
O2—C1—C2—C3	165.7 (6)	C35—C36—C37—C38	0.2 (12)
O1—C1—C2—C3	−14.3 (8)	N3—C37—C38—C39	177.2 (7)
O2—C1—C2—C7	−17.9 (9)	C36—C37—C38—C39	1.0 (12)
O1—C1—C2—C7	162.0 (5)	C35—C34—C39—C38	−1.3 (11)
C7—C2—C3—C4	1.0 (9)	C33—C34—C39—C38	−180.0 (7)
C1—C2—C3—C4	177.5 (6)	C37—C38—C39—C34	−0.5 (11)
C2—C3—C4—C5	−4.0 (10)	Sb2—O7—C40—O8	−2.0 (11)
C3—C4—C5—N1	−177.4 (7)	Sb2—O7—C40—C41	174.4 (5)
C3—C4—C5—C6	4.3 (10)	O8—C40—C41—C46	−12.7 (13)
N1—C5—C6—C7	−180.0 (7)	O7—C40—C41—C46	170.9 (8)
C4—C5—C6—C7	−1.7 (11)	O8—C40—C41—C42	164.4 (9)
C5—C6—C7—C2	−1.3 (10)	O7—C40—C41—C42	−12.0 (12)
C3—C2—C7—C6	1.6 (9)	C46—C41—C42—C43	0.4 (14)
C1—C2—C7—C6	−174.8 (6)	C40—C41—C42—C43	−176.7 (8)
Sb1—O3—C8—O4	−7.2 (7)	C41—C42—C43—C44	1.1 (15)
Sb1—O3—C8—C9	175.6 (4)	C42—C43—C44—N4	−179.0 (10)
O4—C8—C9—C10	−168.2 (6)	C42—C43—C44—C45	−3.8 (15)
O3—C8—C9—C10	9.0 (8)	N4—C44—C45—C46	−179.5 (9)
O4—C8—C9—C14	6.3 (9)	C43—C44—C45—C46	4.9 (15)
O3—C8—C9—C14	−176.5 (5)	C42—C41—C46—C45	0.8 (14)
C14—C9—C10—C11	−1.4 (10)	C40—C41—C46—C45	177.9 (8)
C8—C9—C10—C11	173.2 (6)	C44—C45—C46—C41	−3.5 (15)
C9—C10—C11—C12	−1.4 (11)	C59—Sb2—C47—C48	−112.4 (7)
C10—C11—C12—N2	−178.0 (7)	O5—Sb2—C47—C48	155.0 (6)
C10—C11—C12—C13	4.8 (10)	C53—Sb2—C47—C48	65.9 (7)
N2—C12—C13—C14	177.2 (6)	O7—Sb2—C47—C48	−24.9 (6)
C11—C12—C13—C14	−5.5 (10)	C59—Sb2—C47—C52	68.6 (7)
C12—C13—C14—C9	2.7 (9)	O5—Sb2—C47—C52	−24.0 (7)
C10—C9—C14—C13	0.8 (9)	C53—Sb2—C47—C52	−113.0 (7)
C8—C9—C14—C13	−173.9 (5)	O7—Sb2—C47—C52	156.1 (7)
O1—Sb1—C15—C16	148.7 (5)	C52—C47—C48—C49	1.4 (13)
C21—Sb1—C15—C16	−123.5 (5)	Sb2—C47—C48—C49	−177.6 (7)
O3—Sb1—C15—C16	−34.3 (5)	C47—C48—C49—C50	1.9 (14)
C27—Sb1—C15—C16	58.5 (7)	C48—C49—C50—C51	−6.1 (16)
O1—Sb1—C15—C20	−33.2 (5)	C49—C50—C51—C52	6.9 (16)
C21—Sb1—C15—C20	54.6 (5)	C48—C47—C52—C51	−0.5 (13)
O3—Sb1—C15—C20	143.8 (5)	Sb2—C47—C52—C51	178.5 (7)
C27—Sb1—C15—C20	−123.4 (5)	C50—C51—C52—C47	−3.6 (15)
C20—C15—C16—C17	1.5 (9)	C59—Sb2—C53—C58	1.1 (11)
Sb1—C15—C16—C17	179.5 (5)	O5—Sb2—C53—C58	98.0 (9)
C15—C16—C17—C18	−0.8 (10)	C47—Sb2—C53—C58	−176.8 (8)
C16—C17—C18—C19	−0.3 (11)	O7—Sb2—C53—C58	−89.4 (9)
C17—C18—C19—C20	0.6 (11)	C59—Sb2—C53—C54	−177.0 (7)
C18—C19—C20—C15	0.1 (11)	O5—Sb2—C53—C54	−80.2 (7)
C16—C15—C20—C19	−1.2 (10)	C47—Sb2—C53—C54	5.0 (8)
Sb1—C15—C20—C19	−179.4 (5)	O7—Sb2—C53—C54	92.4 (7)
O1—Sb1—C21—C22	151.9 (7)	C58—C53—C54—C55	5.2 (16)
O3—Sb1—C21—C22	−29.3 (7)	Sb2—C53—C54—C55	−176.5 (9)
C27—Sb1—C21—C22	−118.6 (6)	C53—C54—C55—C56	−4(2)
C15—Sb1—C21—C22	62.7 (7)	C54—C55—C56—C57	2(2)
O1—Sb1—C21—C26	−23.0 (6)	C55—C56—C57—C58	−2(2)
O3—Sb1—C21—C26	155.8 (6)	C54—C53—C58—C57	−4.6 (16)
C27—Sb1—C21—C26	66.5 (6)	Sb2—C53—C58—C57	177.3 (9)
C15—Sb1—C21—C26	−112.2 (6)	C56—C57—C58—C53	3(2)
C26—C21—C22—C23	−0.4 (12)	O5—Sb2—C59—C60	82.4 (9)
Sb1—C21—C22—C23	−175.5 (7)	C47—Sb2—C59—C60	−4.4 (10)
C21—C22—C23—C24	−1.1 (15)	C53—Sb2—C59—C60	177.7 (8)
C22—C23—C24—C25	1.0 (15)	O7—Sb2—C59—C60	−90.5 (9)
C23—C24—C25—C26	0.7 (14)	O5—Sb2—C59—C64	−100.7 (9)
C24—C25—C26—C21	−2.3 (13)	C47—Sb2—C59—C64	172.5 (8)
C22—C21—C26—C25	2.1 (12)	C53—Sb2—C59—C64	−5.4 (11)
Sb1—C21—C26—C25	177.1 (6)	O7—Sb2—C59—C64	86.4 (9)
O1—Sb1—C27—C32	117.0 (5)	C64—C59—C60—C61	−3.9 (19)
C21—Sb1—C27—C32	29.0 (6)	Sb2—C59—C60—C61	173.3 (11)
O3—Sb1—C27—C32	−59.4 (5)	C59—C60—C61—C62	2(2)
C15—Sb1—C27—C32	−153.0 (5)	C60—C61—C62—C63	3(2)
O1—Sb1—C27—C28	−61.0 (6)	C61—C62—C63—C64	−6(2)
C21—Sb1—C27—C28	−148.9 (6)	C60—C59—C64—C63	0.9 (17)
O3—Sb1—C27—C28	122.7 (6)	Sb2—C59—C64—C63	−176.0 (8)
C15—Sb1—C27—C28	29.0 (8)	C62—C63—C64—C59	4.3 (18)

### Hydrogen-bond geometry (Å, °)

**Table:**

*D*—H···*A*	*D*—H	H···*A*	*D*···*A*	*D*—H···*A*
N3—H3B···N2^i^	0.86	2.44	3.245 (11)	156
N3—H3A···O4^ii^	0.86	2.40	3.178 (8)	151
N2—H2B···O2^iii^	0.86	2.22	2.996 (8)	151
N1—H1A···O4^iv^	0.86	2.24	3.046 (8)	156

Symmetry codes: (i) −*x*+1, *y*−1/2, −*z*+1; (ii) *x*, *y*, *z*−1; (iii) −*x*+1, *y*+1/2, −*z*+2; (iv) −*x*+2, *y*−1/2, −*z*+2.
